# Antibiofilm activity of flavonoids on staphylococcal biofilms through targeting BAP amyloids

**DOI:** 10.1038/s41598-020-75929-2

**Published:** 2020-11-03

**Authors:** Leticia Matilla-Cuenca, Carmen Gil, Sergio Cuesta, Beatriz Rapún-Araiz, Miglė Žiemytė, Alex Mira, Iñigo Lasa, Jaione Valle

**Affiliations:** 1 Instituto de Agrobiotecnología (IDAB), CSIC-UPNA-Gobierno de Navarra, Avenida Pamplona 123, 31192 Mutilva, Spain; 2 Navarrabiomed-Universidad Pública de Navarra-Departamento de Salud, IDISNA, 31008 Pamplona, Navarra Spain; 3 Genomics and Health Department, FISABIO Foundation, 46020 Valencia, Spain

**Keywords:** Microbiology, Biofilms

## Abstract

The opportunistic pathogen *Staphylococcus aureus* is responsible for causing infections related to indwelling medical devices, where this pathogen is able to attach and form biofilms. The intrinsic properties given by the self-produced extracellular biofilm matrix confer high resistance to antibiotics, triggering infections difficult to treat. Therefore, novel antibiofilm strategies targeting matrix components are urgently needed. The Biofilm Associated Protein, Bap, expressed by staphylococcal species adopts functional amyloid-like structures as scaffolds of the biofilm matrix. In this work we have focused on identifying agents targeting Bap-related amyloid-like aggregates as a strategy to combat *S. aureus* biofilm-related infections. We identified that the flavonoids, quercetin, myricetin and scutellarein specifically inhibited Bap-mediated biofilm formation of *S. aureus* and other staphylococcal species. By using in vitro aggregation assays and the cell-based methodology for generation of amyloid aggregates based on the Curli-Dependent Amyloid Generator system (C-DAG), we demonstrated that these polyphenols prevented the assembly of Bap-related amyloid-like structures. Finally, using an in vivo catheter infection model, we showed that quercetin and myricetin significantly reduced catheter colonization by *S. aureus.* These results support the use of polyphenols as anti-amyloids molecules that can be used to treat biofilm-related infections.

## Introduction

*Staphylococcus aureus* is one of the leading bacteria causing biofilm‐associated infections related with medical devices^1^. The difficulty of treating such infections is aggravated by the intrinsic resistance to antibiotics given by the biofilm structures produced by these bacteria^2–4^. Therefore, to fight against biofilm-associated infections, investigations have focused on antibiofilm agents targeting the matrix components, so bacteria lose the protection of the matrix, thereby increasing the effectiveness of traditional antibiotic therapies^5–8^.

*Staphylococcus aureus* produces a biofilm matrix composed of exopolysaccharides, surface proteins, extracellular DNA and functional amyloids^9–14^. Two types of amyloids have been described as scaffold biofilm matrix of *S. aureus*. The first group consists of small peptides known as phenol-soluble modulins (PSMs)^13^. These peptides show a tendency to aggregate in amyloid fibers that contribute to the stabilization and maturation of the biofilm structure by preventing disassembly of the biofilm matrix. The second type of staphylococcal amyloids is the group of facultative amyloids. This group comprises surface-associated proteins that adopt an amyloid-like conformation under specific environmental conditions, as the Biofilm Associated Protein (Bap) present in some strains of *S. aureus* and in other coagulase negative staphylococci^15–17^.

Bap is a multidomain secreted protein identified in a transposon-based mutagenesis for the screening of biofilm defective mutants in the bovine mastitis isolate *S. aureus* V329^18^. Bap promotes biofilm formation through a mechanism that is independent of the biofilm associated polysaccharide PNAG. The *bap* gene is frequently identified in mastitis-derived staphylococcal species and all the isolates expressing Bap are strong biofilm formers^19^.

Bap undergoes partial proteolytic cleavage, which releases fragments containing the N-terminal region^17^. This region has a molten globule-like conformation that, when the pH becomes acidic, switches to a β-sheet-rich structure and forms amyloid-like fibers^17^. Others Bap homologs behave the same way. The cleavage of N-terminal domain is enough for the formation of amyloid fibers^17,20^. However, in *S. epidermidis,* the C-repeat domain contains amyloid peptides that are sufficient for spontaneous formation of amyloid fibers^21^. This makes Bap an excellent target for anti-amyloid compounds to reduce staphylococcal biofilm formation.

Polyphenols are natural small molecules composed of one or more aromatic phenolic rings. Because of their antioxidant, anti-platelet and anti-inflammatory effects they appear to have disease-fighting properties including neurological and cardiovascular diseases^22–25^. In some neurodegenerative disorders related to amyloids, polyphenols inhibit toxic amyloids assembly or remove already formed amyloid oligomers and fibrils^26,27^. Polyphenols are also effective against viral and bacterial infections^28–32^. In this context, it has been proposed that the antibacterial mechanisms of polyphenols can be related to their ability to inhibit biofilm formation or to disperse already pre-formed biofilms^33^.

Here, we evaluated the efficacy of a set of polyphenolic molecules with demonstrated anti-amyloid effect on human amyloids to inhibit Bap-mediated biofilm formation. We identified that specifically the flavonoids quercetin, myricetin and scutellarein inhibited biofilms of several *S. aureus* strains and other staphylococcal species expressing Bap. We demonstrated that polyphenols specifically inhibit Bap amyloid-like aggregates. Finally, by using a catheter infection model in mice we showed that quercetin and myricetin reduced catheter-colonization by *S. aureus.* Our results enlighten the potential of polyphenols as promising candidates for inhibiting *S. aureus* biofilms.

## Results

### Screening for polyphenols with antibiofilm activity against amyloid-mediated biofilm

**Figure 1:**
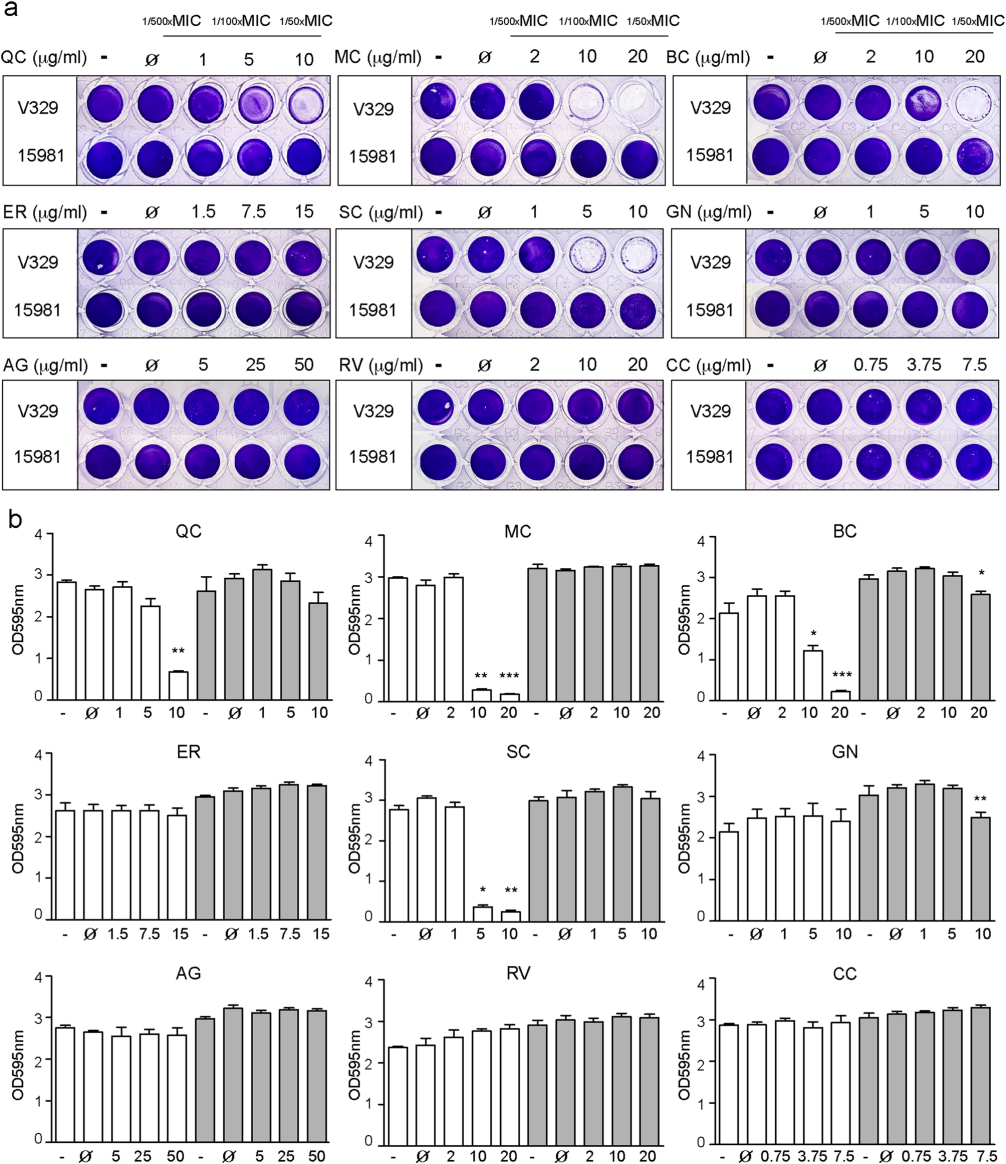
Inhibitory effect of polyphenols in the biofilm formation assay. (**a**) Biofilm formed by *S. aureus* V329 (amyloid-based biofilm) and *S. aureus* 15981 (polysaccharide-based biofilm) in the presence of sub-MICs concentrations of polyphenols (1/500; 1/100; 1/50). The bacterial cells were stained with crystal violet. 2% of DMSO was added as control (Ø). Bacteria cultured in TSB-glu media were used as positive control (−). (**b**) Quantification of the crystal violet after addition of 200 μl of ethanol-acetone and determination of absorbance at 595 nm. *S. aureus* V329 (white bars). *S. aureus* 15981 (grey bars). The error bars represent the standard deviation of the results of three repetitions. Statistical significance was determined with one-way ANOVA followed by multiple comparison test (*P < 0.05; **P < 0.01; ***P < 0.001). *QC* Quercetin, *MC* myricetin, *BC* baicalein, *SC* scutellarein, *ER* eriodictyol, *GN* genistein, *AG* gallic acid, *RV* resveratrol, *CC* curcumin.

We choose a collection of polyphenolic compounds, including flavonoids, stilbenoids and phenolic acids, that have been shown to inhibit fibrillization of human and bacterial amyloids with the aim of identifying polyphenols targeting Bap amyloid-like aggregates (Table 1 and Fig. S1)^27,34^. First, we determined the minimum inhibitory concentrations (MIC) of selected polyphenols against *S. aureus* V329 using the standard broth microdilution method (Table 1). MIC against *S. aureus* V329 were 375 μg/ml for curcumin, 500 μg/ml for quercetin, scutellarein and genistein, 750 μg/ml for eriodictyol, 1000 μg/ml for myricetin, baicalein and resveratrol and 2500 μg/ml for gallic acid. Next, we tested the antibiofilm effect of the polyphenols on *S. aureus* V329 biofilm administered at sub-MIC dosages. As expected none of the polyphenols at the concentrations tested affected bacterial viability (Fig. S2). In order to identify polyphenols that specifically inhibit Bap-dependent biofilm formation, we included the *S. aureus* 15981 strain (*bap* negative), which formed a polysaccharide (PNAG)-dependent biofilm, as a control. After cultivation of *S. aureus* V329 and 15981 strains in the presence of _1/50x_MIC, _1/100x_MIC and _1/500x_MIC of the polyphenols, biofilms were stained with crystal violet. Results showed that myricetin (MC), baicalein (BC), and scutellarein (SC) strongly inhibited biofilm formation of *S. aureus* V329 at _1/50x_MIC (MC 20 μg/ml, BC 20 μg/ml and SC 10 μg/ml) with a percentage of biofilm inhibition of 94% ± 1%, 91% ± 3%, 92% ± 3%, respectively and _1/100x_MIC (MC 10 μg/ml, BC 10 μg/ml and SC 5 μg/ml) with a percentage of biofilm reduction of 92% ± 4%, 69% ± 21%, 84% ± 1%, respectively (***P < 0.001, **P < 0.01, *P < 0.05) (Fig. 1A,B). Quercetin (QC) significantly reduced biofilm at _1/50x_MIC (10 μg/ml) with a percentage of biofilm inhibition of 75 ± 2 (**P < 0.01). Only genistein (GN) (10 μg/ml) and baicalein (BC) (20 μg/ml) significantly reduced biofilm of *S. aureus* 15981 (**P < 0.01, *P < 0.05) (Fig. 1A,B). With these results, we have established that the minimal biofilm inhibitory concentrations (MBIC) of polyphenols to inhibit biofilms in which Bap is a matrix component are 10 μg/ml for QC, 10 μg/ml for MC and 5 μg/ml for SC, respectively.

**Table 1:** Polyphenols used in this study.

	MIC (μg/ml)	MBIC-Bap (μg/ml)
**Flavonoids**		
Quercetin (QC)	500	10
Myricetin (MC)	1000	10
Baicalein (BC)	1000	10
Scutellarein (SC)	500	5
Eriodictyol (ER)	750	–
Genistein (GN)	500	–
**Phenolic acids**		
Gallic acid (AG)	2500	–
**Stilbenoids**		
Resveratrol (RV)	1000	–
**Others**		
Curcumin (CC)	375	–

*MIC* Minimum inhibitory concentration, *MBIC-Bap* Minimum biofilm inhibitory concentration.

**Figure 2:**
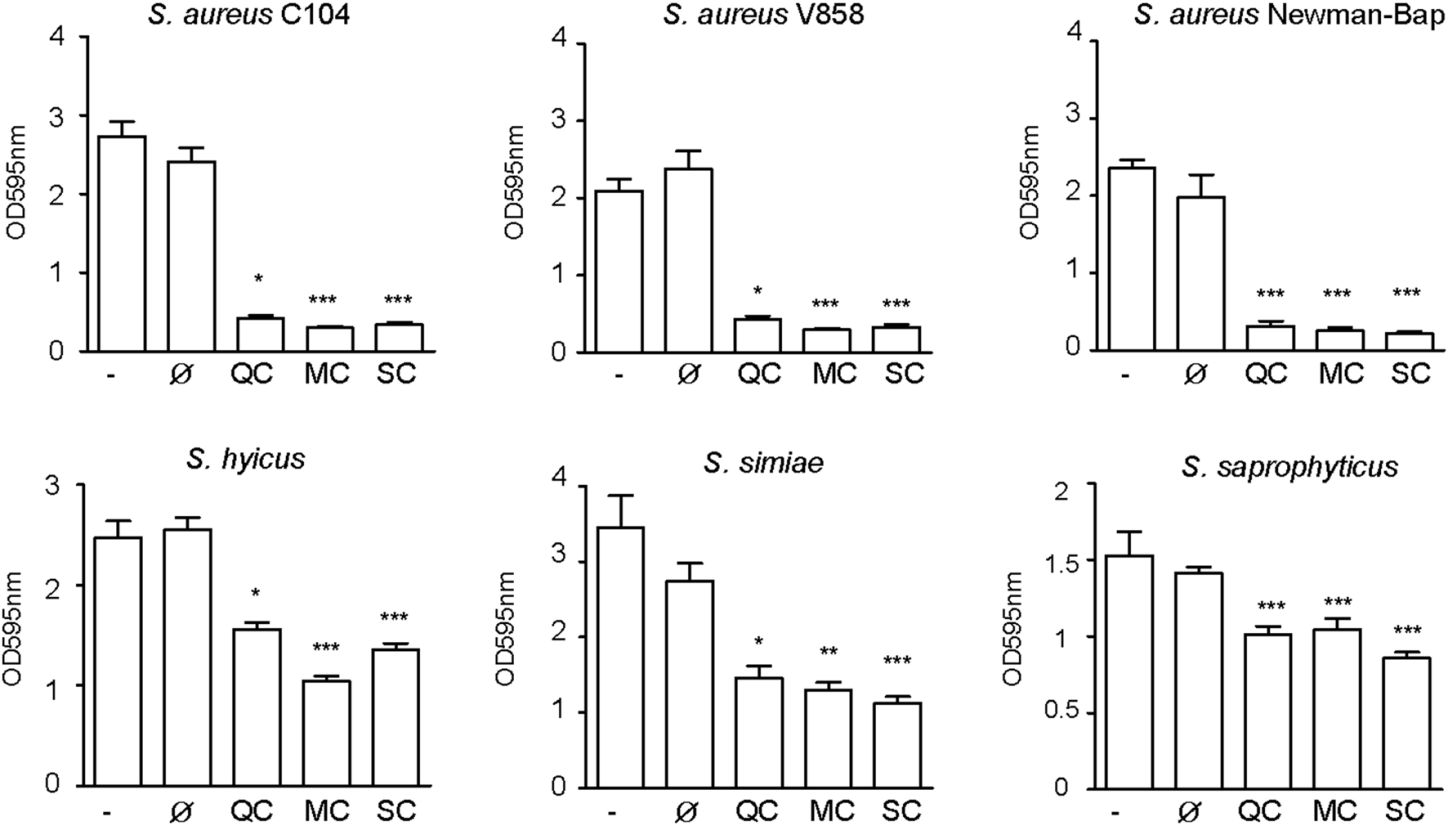
Antibiofilm activity of polyphenols against 3 non-related *S. aureus* strains that express Bap (C104, V858 and Newman-Bap) and other staphylococcal species (*S. hycus*, *S. simiae* and *S. saprophytycus*) that form Bap-dependent biofilms. MBIC were used as 10 μg/ml for quercetin (QC), 10 μg/ml for myricetin (MC) and 5 μg/ml for scutellarein (SC). 2% of DMSO was added as control (Ø). Bacteria cultured in TSB-glu media were used as positive control (−). Crystal violet was quantified by measuring absorbance at 595 nm. Statistical significance was determined with one-way ANOVA followed by multiple comparison test (*P < 0.05;**P < 0.01; ***P < 0.001).

To confirm that QC, MC and SC inhibited Bap-dependent biofilm formation we analyzed the antibiofilm activity of the polyphenols against 3 non-related *S. aureus* strains that express Bap (Newman-Bap, C104 and V858). Quantification of the biofilm formed by these strains after incubation with polyphenols showed a significant reduction of the bacteria adhered to the microtiter plate (Fig. 2) (***P < 0.001). We next analyzed the effect of the polyphenols against the biofilm formed by Bap-producing coagulase negative staphylococcal species *S. hyicus*, *S. saprophyticus* and *S. simiae*. The results showed that QC, MC and SC reduced biofilm formed by the staphylococcal species, even though their effect was not as strong as the effect observed on the *S. aureus* strains (*P < 0.05;**P < 0.01;***P < 0.001) (Fig. 2). Taken together, these results indicated that the QC, MC and SC polyphenols prevent biofilm formation in Bap-producing staphylococcal strains.

### Real-time analysis of the antibiofilm effect of the polyphenols on *S. aureus*

**Figure 3:**
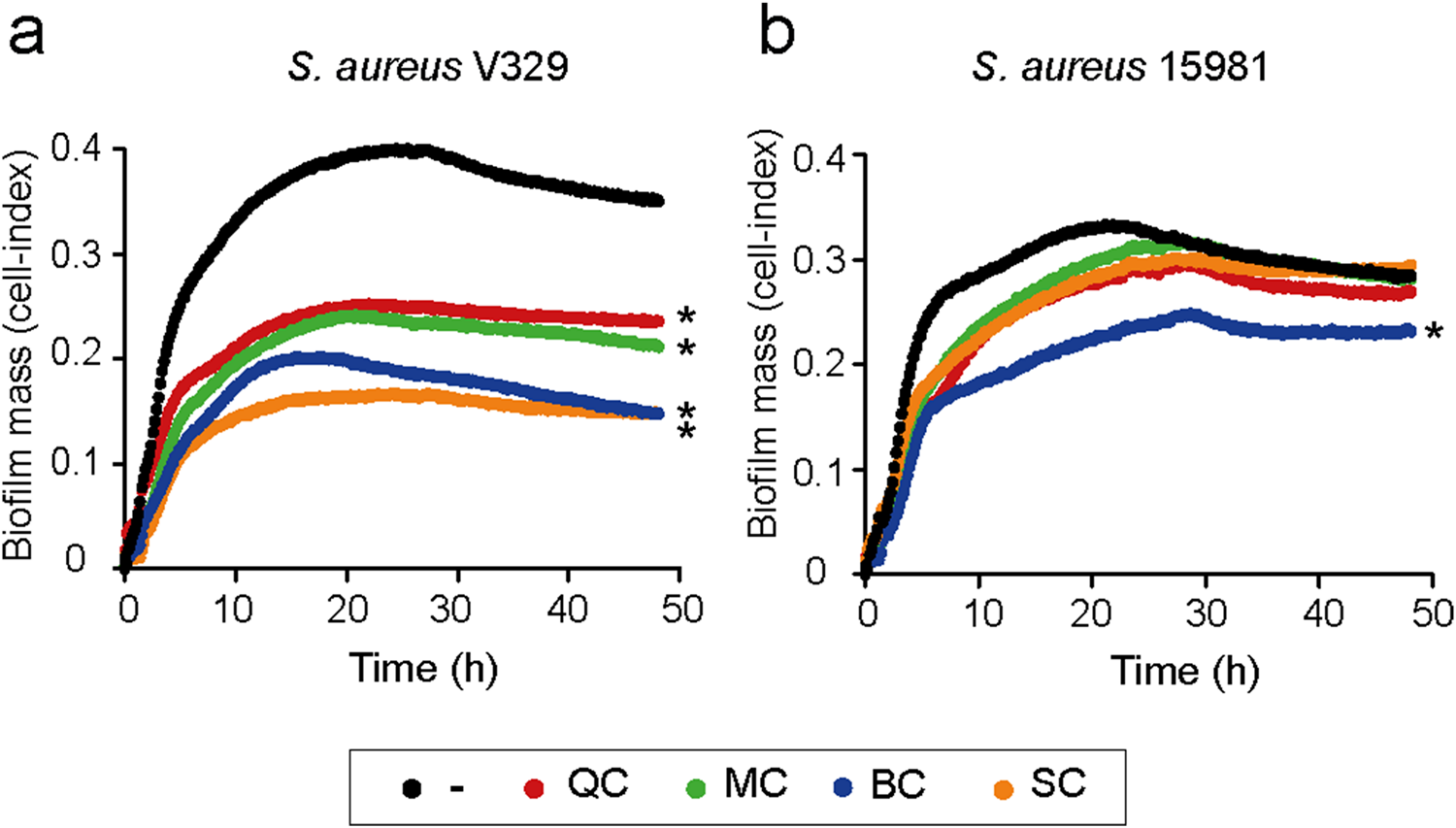
Biofilm growth of *S. aureus* V329 (**a**) and 15981 (**b**) in the presence of polyphenols. Graphs show total biofilm mass through time, as determined by impedance measurements in the absence (black line) and presence of quercetin (QC) 10 μg/ml, myricetin (MC) 10 μg/ml, scutellarein (SC) 5 μg/ml and baicalein (BC) 10 μg/ml. Quantification of biofilm growth was recorded every 10 min at 37 °C. Each line represents the mean of three biological replicates. For statistical differences in biofilm CI values, regression analysis was assessed by a linear model between 15 and 25 h of biofilm growth, using the lm library in the R statistical package version 1.0.7.1. (*P < 0.05). SDs are not shown for clarity.

We used the xCELLigence impedance system to quantitatively evaluate the antibiofilm effect of the polyphenols on the *S. aureus* strains V329 and 15981 on real-time. When the polyphenols QC, MC, BC and SC were added into the culture medium at the MBIC (10 μg/ml for QC, MC, BC, and 5 μg/ml for SC, respectively), a significant reduction in the biofilm mass of *S. aureus* V329 was observed. Specifically, QC, MC, BC and SC inhibited *S. aureus* V329 biofilm formation by 37% ± 0.25%, 39% ± 0.09%, 58% ± 0.19% and 50% ± 0.23%, respectively (Fig. 3A). Interestingly, inhibition of the biofilm formation capacity persisted after 48 h of growth, with significant reductions in the Cell Index (CI), a measure of total biofilm mass, of 33% ± 0.22% for QC, 39% ± 0.25% for MC, 58% ± 0.1% for BC and 58% ± 0.08% for SC (*P < 0.05) (Fig. 3A). In the case of *S. aureus* 15981, the addition of QC, MC and SC resulted in a biofilm growth delay that did not cause a significant reduction in the CI values after 48 h of growth (Fig. 3B). Only BC inhibited biofilm formation of 15981 by approximately 27% ± 0.37% and 18% ± 0.42% at 24 h and 48 h of biofilm growth respectively (Fig. 3B). Altogether, these results confirm that the polyphenols QC, MC and SC have a strong and specific capacity to inhibit Bap mediated biofilms, while BC is capable to prevent both the proteinaceous and polysaccharidic biofilm formation process.

### Effect of polyphenols on Bap expression

**Figure 4:**
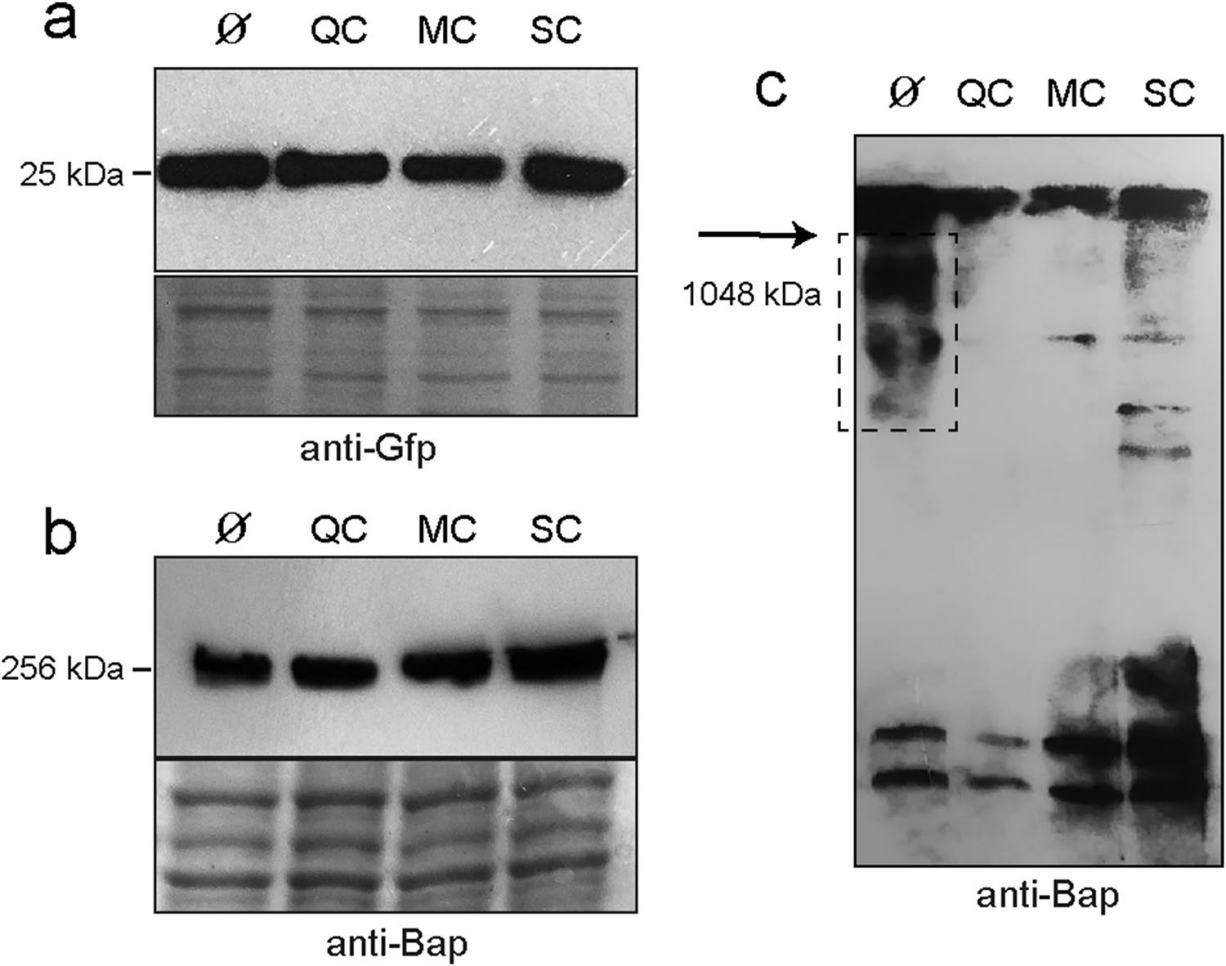
Western immunoblot showing the effect of polyphenols on the expression and aggregation of Bap. (**a**) GFP protein levels of *S. aureus* V329 with pCN52-P_*bap*_:GFP plasmid in presence of MBIC of : 10 μg/ml for quercetin (QC), 10 μg/ml for myricetin (MC) and 5 μg/ml for scutellarein (SC). (**b**) Bap protein levels of *S. aureus* V329 in presence of MBICs of polyphenols. (**c**) Native immunoblotting of cell surface extracts *S. aureus* V329 cultured in presence of MBICs of polyphenols. Bap-related insoluble aggregates are indicated by a dashed line box. Untreated bacterial cells were used as positive control (Ø).

We next evaluated whether the expression of the *bap* gene was affected by the presence of the polyphenols. We constructed a *S. aureus* V329-P_*bap*_ strain, harboring a transcriptional fusion between *bap* promoter and *gfpmut2* expressed in plasmid pCN52. Western blot using anti-GFP specific antibodies revealed that the addition of MBIC of QC, MC and SC did not affect *bap* promoter expression (Fig. 4A). Alternatively, polyphenols could inhibit biofilm development by reducing the amount of Bap anchored to the bacterial surface. Thus, we analyzed the levels of the Bap protein of the cell surface fraction of *S. aureus* V329 grown until exponential phase in the presence of MBICs of the polyphenols. Western immunoblotting using anti-Bap antibodies showed the same levels of Bap in all the samples (Fig. 4B). Since polyphenols did not affect Bap expression, we hypothesized that they might inhibit biofilm formation by physically interfering with the capacity of Bap to form amyloid aggregates. To evaluate this hypothesis, we monitored Bap aggregation using native gel electrophoresis of *S. aureus* V329 cells in the presence of QC, MC and SC, grown until late stationary phase which is the growth stage where the Bap amyloid-like aggregates are formed ^17^. Western immunoblotting showed the presence of several bands of high molecular weight (~ 1000 kDa) corresponding to Bap aggregates in the absence of polyphenols. The amount of aggregates was lower when bacteria were cultured in the presence of the polyphenols (Fig. 4C). These data suggest that polyphenols affected the formation of Bap amyloid-like aggregates.

### Polyphenols interfere with Bap polymerization process

**Figure 5:**
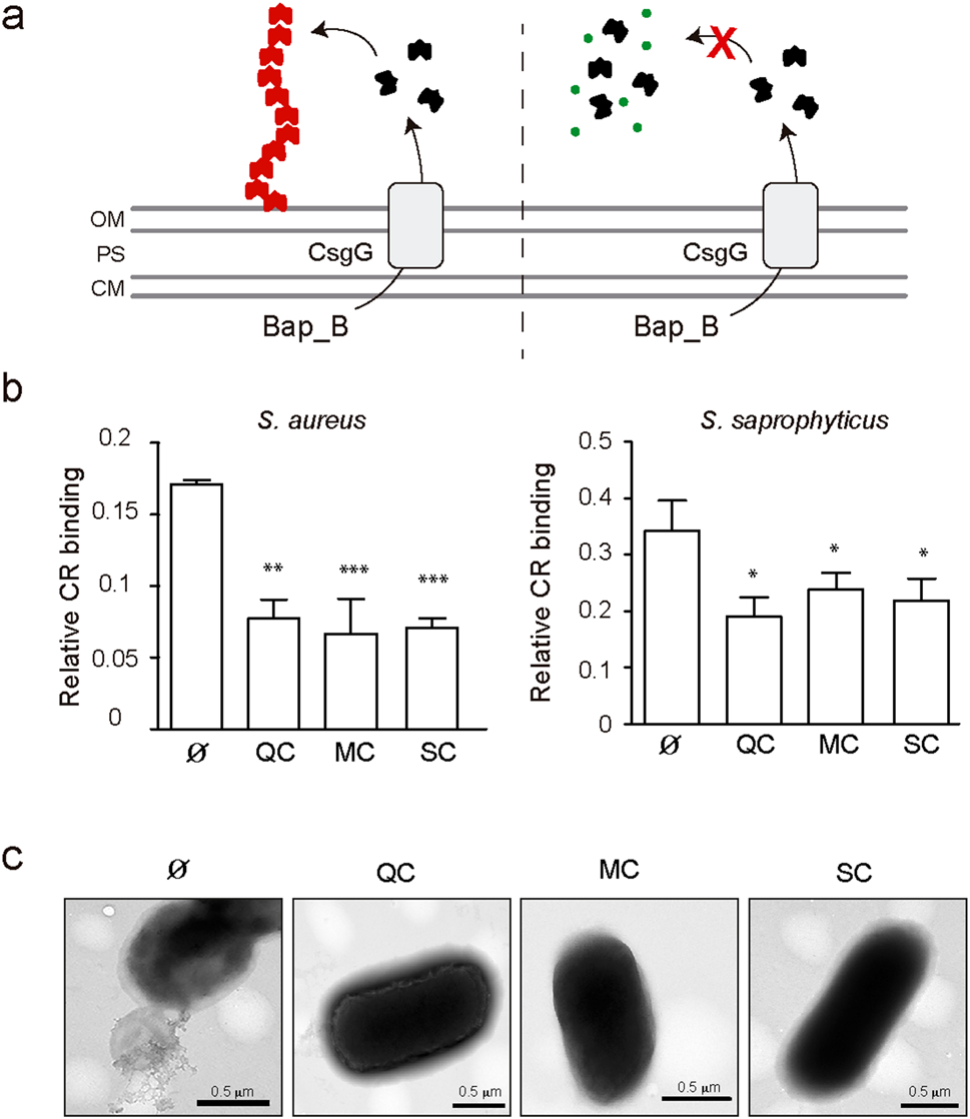
Assembly of Bap_B using the curli-dependent amyloid generator (C-DAG) in the presence of polyphenols. (**a**) Representative scheme of C-DAG. Bap_B domain is cloned under the control of the inducible PBAD promoter and the signal sequence of CsgA. CsgG is expressed under the control of an IPTG-inducible promoter and forms a pore in the OM for externalization of Bap_B. Bap_B forms amyloid-like fibers (left panel). Polyphenols (green circles) can interrupt the assembly of Bap_B into amyloid fibers (right panel). Outer membrane (OM), cytoplasmic membrane (CM), periplasmic space (PS). (**b**) Quantification of CR bound to *E. coli* cells expressing Bap_B from *S. aureus* and *S. saprophytycus* in the presence of MBIC of polyphenols. Ø, without polyphenols. The relative effect of the polyphenols was calculated as OD_500nm_ Bap_B − OD_500nm_ Bap_A. Bars represent standard deviations of the results of five independent experiments (n = 5). Statistically significant differences were determined using Mann–Whitney test *P < 0.05. (**c**) Transmission electron micrographs of negatively stained fiber-like structures formed by *E. coli* cells that express *S. aureus* Bap_B. Extracellular fibrous structures are not observed in the presence of polyphenols.

To test whether the polyphenols hinder Bap capacity to generate amyloid aggregates, we used the curli-dependent amyloid generator (C-DAG) system^35^. When the N-terminal domain of the Bap protein (Bap_B amyloid domain) is heterologous expressed and exported in the *E. coli* strain of C-DAG system it self-assembles into amyloid-like structures (Fig. 5A)^17^. Aggregates are detected by analyzing the capacity of the resulting strain to bind Congo Red dye (CR). To remove the color background of polyphenols, relative CR binding was determined by subtracting the CR value of *E. coli* cells expressing Bap_A, a non-amyloidogenic domain. As shown in Fig. 5B, the relative CR binding of *E. coli* cells cultured in the presence of MBIC of polyphenols (10 μg/ml for QC, 10 μg/ml for MC and 5 μg/ml for SC) were significantly lower (**P < 0.01) than the CR binding of *E. coli* cells grown in the absence of polyphenols. Polyphenols caused no inhibition of *E. coli* growth (Supplementary Fig. S3). Similar results were obtained when polyphenols were added to *E. coli* cells expressing Bap_B domain of *S. saprophyticus* (Fig. 5B). Furthermore, electron microscopy analysis of the *E. coli* strain exporting the *S. aureus* Bap_B domain revealed the presence of extracellular fibrous aggregates surrounding the cells. However, fibrillar structures were not observed in *E. coli* strain cultured in the presence of polyphenols (Fig. 5C). These results confirmed that polyphenols inhibit bacterial amyloid formation.

### The polyphenols hinder the formation of rBap_B amyloid polymerization

**Figure 6:**
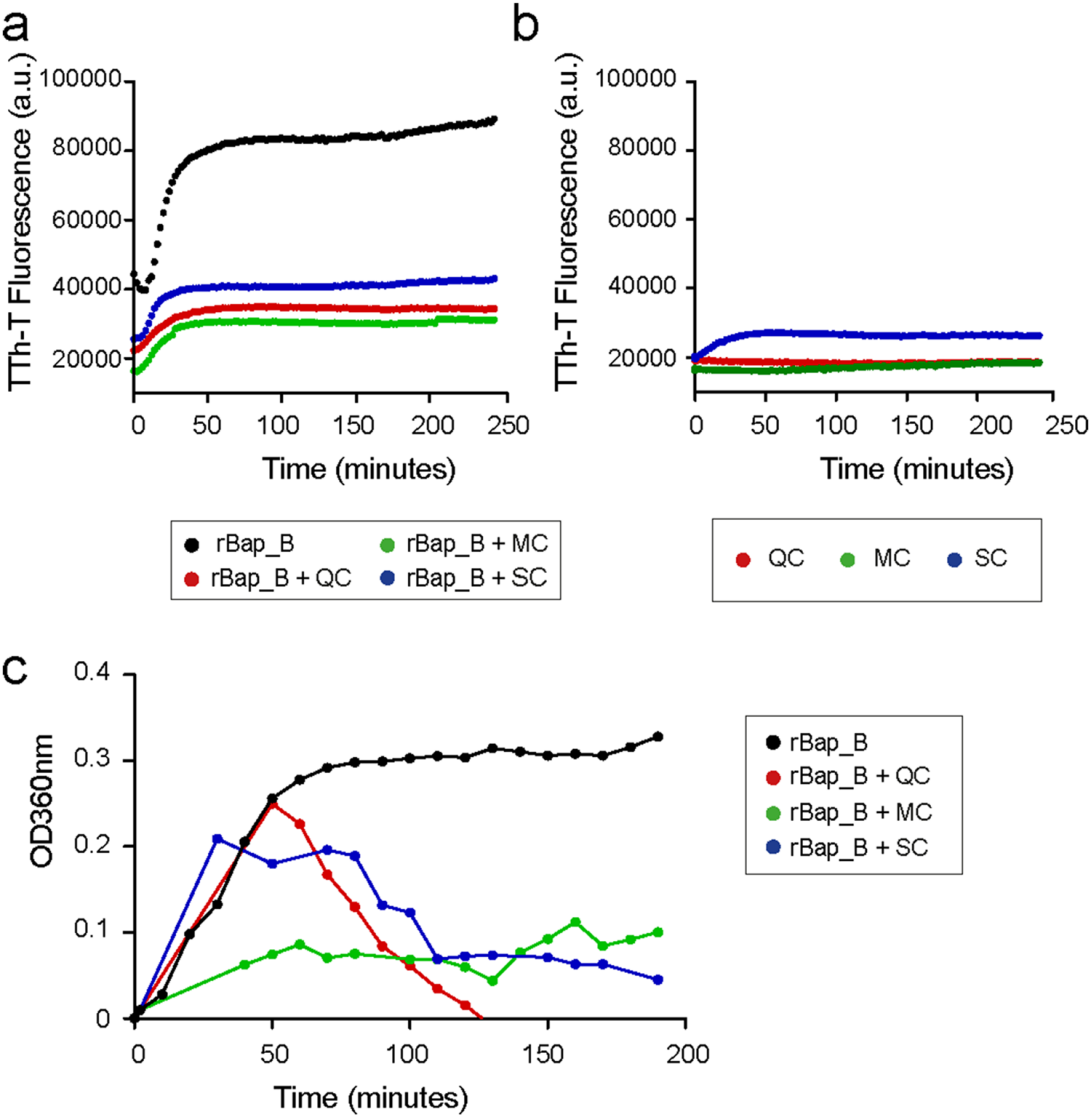
Polyphenols inhibit aggregation of a recombinant rBap_B. (**a**) Kinetic aggregation of recombinant rBap_B protein at 0.1 mg/ml in presence of 200 µM of the polyphenols in phosphate-citrate buffer pH 4.4. Th-T amyloid-dye was added and emission spectra were recorded in the range of 460–600 nm. (**b**) The basal fluorescence of polyphenols themselves were recorded. (**c**) Kinetic aggregation assays of rBap_B in the in presence of polyphenols, followed by solution turbidity. Turbidity was measured as absorbance at 360 nm every 10 min. Black dots: positive aggregation control (only rBap_B).

To investigate whether the polyphenols modulate Bap amyloid aggregation independently of additional bacterial factors, we measured the capacity of a purified recombinant protein comprising exclusively the B region of Bap (rBap_B) to bind Th-T in the presence of polyphenols. The results showed that rBap_B polymerization was significantly inhibited in the presence of polyphenols (Fig. 6A). None of the polyphenolic molecules displayed background fluorescence in these conditions when they were assayed without the rBap_B protein (Fig. 6B).

To confirm that the polyphenols identified were true aggregation inhibitors and not a misleading artefact produced by the displacement of Th-T from amyloid by polyphenols, we used a turbidimetric assay^36^. Aggregation of rBap_B gives rise to high molecular weight aggregates that scatter light and increase the turbidity of the solution. Recombinant rBap_B was incubated in the presence of each polyphenolic compound QC, MC and SC for 3 h, and the absorbance at 360 nm was measured over time. The three polyphenols reduced the building of turbidity in the rBap_B solution and were considered to be aggregation inhibitors (Fig. 6C).

### QC and MC reduce *S. aureus* colonization of subcutaneous catheters

**Figure 7:**
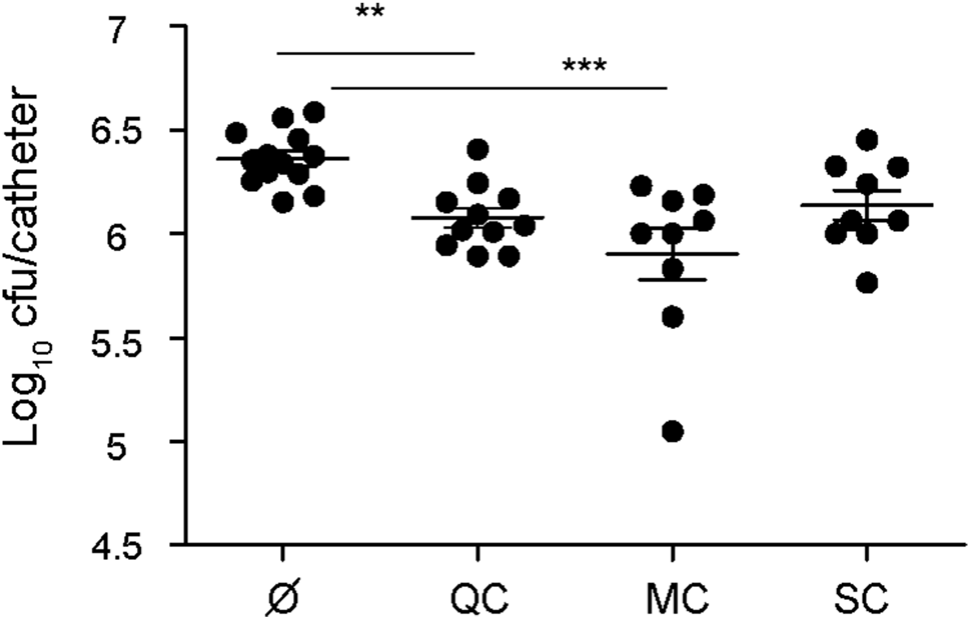
Efficacy of polyphenols in *S. aureus* biofilm infection model. Two catheters infected with *S. aureus* strain V329 were implanted at the subscapular space of groups of mice (n = 6), followed by subcutaneous administration of quercetin (QC) 100 mg/kg, myricetin (MC) 20 mg/kg, scutellarein (SC) 17.5 mg/kg post-infection and repeated every day for 10 days. Animal were killed and bacteria were recovered from implanted catheters and counted. The plots display values obtained from individual catheters and the mean is represented by horizontal bars. Statistical significance was determined with one-way ANOVA followed by Dunn’s multiple comparison test comparing to mice that received DMSO as control, Ø. (**P < 0.01; ***P < 0.001).

Bap-mediated biofilm is important for the colonization and persistence of indwelling medical devices by *S. aureus *in vivo^17^. Therefore, we decided to investigate whether the polyphenols were able to reduce *S. aureus* colonization of implanted subcutaneous catheters in vivo. For this purpose, polyphenols were administered subcutaneously after catheter implantation and repeated daily. At day 10, all animals were killed and catheters were extracted for colony-forming unit (CFU) counting. The results showed that treatment with QC and MC significantly reduced the number of bacteria attached to the catheter with percentage reduction of 53% ± 13% and 54% ± 17%, respectively (**P < 0.01;***P < 0.001) (Fig. 7). Treatment with SC, slightly reduced the catheter colonization by *S. aureus*, but the reduction was not statistically significant.

## Discussion

Several therapeutic alternatives have been proposed to treat medical device-related infections. These include the use of effective antibiofilm molecules or substances that dissolve the biofilm matrix^9^. Here, we show that the polyphenols quercetin (QC), myricetin (MC) and scutellarein (SC) efficiently inhibit the staphylococcal biofilm matrix by specifically targeting Bap-like amyloids. Particularly, MC and its derivative QC acts as molecules that reduces *S. aureus* Bap-mediated biofilms using an in vivo catheter infection mice model.

Polyphenols are a group of biologically active secondary metabolites of plants able to disturb the polymerization of human amyloids responsible for important disorders by inhibiting toxic amyloid formation and/or remodeling or degrading amyloid oligomers and fibrils^26,27^. The structure similarities between human and bacterial amyloids have led to think that polyphenols may affect amyloid-based biofilms. One example is the polyphenol epigallocatechin-3-gallate (EGCG) that has been shown to inhibit the oligomerization of multiple human amyloids such as amyloid-β^37–39^, α-synuclein^38,40–42^, islet amyloid polypeptide^43,44^, huntingtin^45^, tau protein^46^ and prion proteins^47^, but it is also considered an effective agent for remodeling of bacterial amyloid fibrils as FapC in *Pseudomonas* sp*.* strains^48^, the amyloid proteins P1, WapA and Smu_63c of *Streptococcus mutans*^49^, CsgA in Enterobacteria^50,51^. In staphylococcal species EGCG prevents phenol-soluble modulins (PSM) fibril formation and hinders Bap mediated biofilms^17,52,53^. These findings support the hypothesis that polyphenols that are capable to inhibit human amyloids can be used to prevent staphylococcal biofilm formation by avoiding Bap protein to form functional amyloid-like fibers.

Our results showed that QC, MC and SC inhibit *S. aureus* biofilm formation by targeting Bap amyloids without affecting Bap expression. Although the exact mechanism by which these polyphenols reduce Bap aggregation remains unknown it is likely that a direct interaction of polyphenols with different forms of the oligomers could impede the polymerization of the fibers. Our Th-T assay confirms that rBap_B aggregation kinetics follows a sigmoidal curve with a small lag phase that reflects a rapid nucleation-dependent growth and flavonoids disrupt this mechanism. These evidences demonstrate that QC, MC and SC likely bind monomeric Bap_B or early oligomers preventing its oligomerization into amyloid fibrils and thus, inhibiting *S. aureus* biofilm formation.

It is likely that the reason for the inhibitory process of Bap mediated biofilms between the different polyphenols is related to the polyphenolic structure. The aromatic rings of polyphenols may interact and compete with the prevalent aromatic residues (Phe and Trp) of the amyloids and block the self-assembly process and the fiber formation. Furthermore, the hydrophobic hydroxyl groups of polyphenols could bind to the hydrophobic residues of amyloid proteins and inhibit amyloidogenesis^54^. Thus, the selected flavonoids have 3 phenolic rings, at least 3 hydroxyl groups and in all cases 2 of them are vicinal, which are reported to have higher inhibitory effects on human amyloid proteins^34^. Interestingly, QC and MC are closely related in structure, with only one hydroxyl group of difference. The same occurs between SC and BC (Fig. S1). This may explain why these four polyphenols had the strongest inhibitory effect against *S. aureus* V329 biofilm. It is noteworthy that BC is able to inhibit both amyloid-like and PNAG-dependent biofilms. On the one hand, BC showed a significant reduction of an amyloid-like biofilm at sub-MIC concentrations of 20 μg/ml and 10 μg/ml (_1/50x_MIC and _1/100x_MIC). On the other hand, it also reduced PNAG-dependent biofilm of *S. aureus* 15981 at a concentration of 20 μg/ml. Although we discarded this polyphenol from this study due to its broad action, the inhibitory effect of BC on the two types of biofilms make it a promising candidate polyphenol and it would be worth undertaking more detailed studies in the future.

The effect of QC, MC and SC on Bap amyloid assembly was analyzed using the C-DAG system, which provides several advantages: (i) it enables the study of molecule modulators of amyloid aggregation under a uniform set of conditions, (ii) it allows to screen the effect of anti-amyloid molecules directly against the amyloid domain of a protein (Bap_B domain), (iii) it allows independence from the genetic background of *S. aureus* since Bap protein is heterologous expressed in an *E. coli* strain. We also used the C-DAG system to expand the anti-amyloid effect of the polyphenols to the amyloid domains of Bap homologs from staphylococcal coagulase negative. Therefore, this methodology could be used to extend the possible antibiofilm effect of polyphenols on other proteins of the BAP family or other amyloid proteins.

Microbial amyloids are potential contributors to the infectious process^15,55–57^. In the present study we have shown that QC and MC, which are closely related in structure, effectively reduced *S. aureus* colonization in vivo in a murine subcutaneous catheter infection model. The administration of these molecules may allow to minimize not only the effective doses of each agent but also fight the emergence of drug-resistant bacteria. Bap of *S. aureus* belongs to the Biofilm Associated Protein (BAP) family. Homologous Bap proteins are present in many pathogenic bacteria. Our previous results suggest that the mechanism of amyloid-like aggregation might be widespread among these proteins^17,20^. This assumption opens an exciting line of research to consider BAP proteins as molecular targets of polyphenols to fight against biofilm related infections.

## Methods

### Bacterial strains and growth conditions

*S. aureus* V329 and 15981 were used as model strains. *S. aureus* V329 isolated from a bovine mastitis expresses Bap and forms amyloid-like biofilms. Deletion of *bap* gene abolishes biofilm formation in this strain^18^. *S. aureus* 15981 is a *bap* negative clinical isolate that forms polysaccharide (PNAG) mediated biofilms^58^. Deletion of *ica* genes responsible of PNAG synthesis, abolish biofilm formation in this strain. As Bap positive strains we used: *S. aureus* Newman_Bap^59^; *S. aureus* C104^60^; *S. aureus* V858^61^; *S. saprophyticus B20080011225, S. hyicus 12* and *S. simiae CCM7213*^17^. Staphylococcal strains were cultured in trypticase soy broth supplemented with glucose (0.25%, w/v) (TSB-glu).

### Polyphenols

The polyphenols listed in Table 1 were obtained from Sigma-Aldrich (purity ≥ 98%; St. Louis, USA) and Quimigen. The stock and work solutions of polyphenols were prepared in a mixture of dimethyl sulfoxide (DMSO). The DMSO that remained after dilution of polyphenol work solutions into culture broth, even at the highest tested concentration (2%, v/v) caused no inhibition of bacterial growth (Supplementary Fig. S3).

### Determination of minimal inhibitory concentration (MIC)

The MIC values of the polyphenols against *S. aureus* V329 were determined using the twofold serial microdilution method^62^. The tested compounds were added to a 96-well plate containing sterile TSB-glu medium reaching final concentrations of 5–5000 μg/ml. Bacterial inoculum in the medium without the tested compound served as a positive control, whereas the tested compounds in medium without bacteria served as negative control. Following a 24 h incubation, the bacterial growth was evaluated by measuring the turbidity in each well through spectrophotometric analysis (600 nm). The lowest concentration that did not produce detectable absorbance values until the end of incubation was considered as the MIC. All tests were repeated three times to verify the reproducibility of results. Subsequently sub-MICs values of _1/50x_MIC, _1/100x_MIC and _1/500x_MIC were determined for their capacity to inhibit biofilm formation.

### Biofilm inhibition assay

Overnight cultures with OD_600_ = 5 (~ 7.5 × 10^9^ CFU/ml) were diluted 1:40 in TSB-glu medium with sub-MIC dosages of the polyphenols. To exclude a DMSO effect on biofilm formation, overnight culture was diluted in TSB-glu medium with 2% DMSO. Bacteria culture in TSB-glu media was used as positive control. 200 μl of this cell suspension were inoculated in 96-wells polystyrene microtiter plates. After 18 h of incubation at 37 °C, wells were washed with sterile phosphate-buffered saline (PBS) and stained with 0.1% crystal violet. The optical density at 595 nm of the solubilized colorant was determined using a microplate reader (Multiskan EX; Lab-Systems). Each assay was performed in triplicate. Statistical significance differences were determined with one-way ANOVA followed by Dunn’s multiple comparison test: *P < 0.05, **P < 0.01, ***P < 0.001.

### Real-time biofilm growth analysis

Continuous real-time biofilm monitoring was performed using xCELLigence RTCA (Real-Time Cell Analysis) equipment (Agilent). This assay is based on the ability of bacteria to impede electric current when they attach and grow on the gold electrodes placed at the bottom of 96-well microtiter plates. Bacterial biofilms grown in microtiter plates with electrodes at the bottom of wells impedes the flow of electrical current. The impedance is expressed as Cell Index (CI) values, which correlate with the total biofilm mass. The experiments were performed as described previously^63,64^. Briefly, 100 µl of each polyphenol (Table 1) diluted in TSB-glu was used as background for impedance measurements (reaching final concentrations of 10 µg/ml for QC, BC and MC and 5 µg/ml for SC, respectively). Further, 100 µl of *S. aureus* V329 and 15981 bacterial suspensions (OD_600_ = 0.175) were added into the corresponding wells of a 96-well plate, reaching an initial optical density of 0.0875 (10^7^–10^8^ cells, depending on the strain). Impedance data were registered at 10-min intervals for 50 h at 37 °C. Three replicates of each polyphenol and their respective controls were included in each experiment, which was repeated three times. In order to study statistical differences in biofilm CI values, regression analysis was assessed by a linear model between 15 and 25 h of biofilm growth, using the lm library in the R Statistical Package version 1.0.7.1 (Calgano, 2013) (accessed in November, 2019).

### SDS-PAGE and immunoblot assays

Overnight cultures were diluted 1:100 in media supplemented with MBIC of polyphenols (Table 1). Bacteria were grown in microtiter plate under static conditions (biofilm formation conditions) until exponential (OD_600nm_ = 0.8) and stationary phase (OD_600nm_ = 5). For surface protein extracts, cells were resuspended in 100 μl of PBS containing 26% w/v raffinose (Sigma) and 3 μl of 1 mg/ml lysostaphin and incubated 2 h at 37 °C. For total protein extracts, cells were resuspended in 1 ml of PBS and lysed using FastPrep-24 instrument (MP Biomedicals). Supernatants were quantified using Bio-Rad protein assay kit. 15 μg and 1 μg of proteins were load on acrylamide gels (7.5% and 12% separation gels; 4% stacking gels) and were stained with 0.25% Coomassie brilliant blue R250 as loading controls. For native gels, surface proteins were separated in Criterion 3–8% XT Tris–acetate gels and Tris/glycine running buffer (BioRad). Bap protein was detected using rabbit polyclonal Anti-BapB diluted 1:5000. Peroxidase-conjugated goat anti-rabbit immunoglobulin G diluted 1:5000 (Thermo) was used as a secondary antibody. For detection of GFP, mouse monoclonal anti-GFP diluted 1:5000 (Clontech) and peroxidase-conjugated goat anti-mouse immunoglobulin G diluted 1:2500 (Pierce) were used as primary and secondary antibodies, respectively. Full-length gels and blots are included as Supplementary Fig. S4.

### Inhibition of Bap polymerization with the C-DAG system

We used *E. coli* VS39 strain with the pExport plasmid containing region Bap_B of *S. aureus* and *S. saprophyticus* fused to ssCsgA^17,35^. Strains were grown overnight in LB supplemented with ampicillin, 100 µg/ml and chloramphenicol, 20 µg/ml. The overnight cultures were diluted to an OD of 0.01 in LB supplemented with the same antibiotics. To induce CsgG, isopropyl B-d-thiogalactopyranoside (IPTG) was added to a final concentration of 1 mM. Cultures were incubated at 37 °C and 200 rpm until OD_600nm_ reached 0.03 and then L-arabinose was added to a final concentration of 0.2% (w/v). Two milliliters of the cultures were transferred to a 24-well plate containing the MBICs of the polyphenols: QC 10 μg/ml, MC 10 μg/ml and SC 5 μg/ml (Table 1). Bacteria were grown overnight at 37 °C under static conditions. Bap_B aggregation capacity was assessed by evaluating the amount of Congo Red (CR) dye bound to the cells. Bacterial pellets were stained with Congo Red solution (1.2 ml/l of a 0.8% (w/v) for 10 min. Colorant was solubilized in ethanol:acetone (80:20 v/v) during 1 h at 44 °C. The relative effect of the polyphenols was calculated as OD_500nm_ Bap_B—OD_500nm_ Bap_A (negative control). Independent experiments were performed five times. Statistical significance differences were determined using Mann–Whitney test *P < 0.05.

### Microscopy analysis

For transmission electron microscopy, cells were grown overnight in the corresponding tested conditions, washed twice with PBS and then fixed with 2% (v/v) paraformaldehyde (Sigma) for 1 h at room temperature^48^. Formvar/carbon-coated nickel grids were deposited on a drop of fixed sample during five minutes and rinsed three times with PBS. Negative staining was performed using 2% (v/v) uranyl acetate (Agar Scientific, Stansted, UK). Observations were made with a JEOL 1011 transmission electron microscope.

### Thioflavin T binding

rBap_B protein at 0.1 mg/ml was incubated in phosphate-citrate buffer pH 4.4. in a 96-well plate Nunc™ F96 MicroWell™ (ThermoFisher Scientific) with 200 µM of each polyphenol (Table 1). Before measuring thioflavin-T (Th-T) was added at the final concentration of 25 µM. Fluorescence emission spectra were recorded in the 460–600 nm range with an excitation wavelength of 445 nm using a 5 nm slit width for excitation and emission at 25 °C in the multi-mode reader Synergy H1 Hybrid Multi-Mode Reader.

### Turbidity test

Turbidity test was used to discriminate false positives identified at the Th-T assay. For this purpose, rBap_B at 0.1 mg/ml was incubated in phosphate-citrate buffer pH 4.4 in the presence of polyphenols: QC 10 μg/ml, MC 10 μg/ml and SC 5 μg/ml. Mixture was incubated in a 96-well plate at 37 °C for 200 min and the absorbance was measured every 10 min at 360 nm. Every polyphenol was tested in triplicate. Recombinant protein without polyphenols was used as a positive control.

### Murine model of catheter-associated biofilm formation

Bacteria grown overnight on TSA plates were resuspended in PBS to an OD 0.2 (10^8^ CFU/ml). Groups of five-weeks old ICR female mice (n = 6) (Envigo) were anesthetized with isoflurane. Two 19 mm intravenous catheters (24G; B. Braun) were implanted into the subcutaneous interscapular of each mouse and inoculated with 100 µl of *S. aureus* V329. Polyphenols were administered subcutaneously at the interscapular space during 10 days at non-toxic concentrations of QC 100 mg/kg, MC 20 mg/kg and SC 17.5 mg/kg^65–68^. After 10 days, animals were anesthetized by isoflurane inhalation followed by euthanatized by cervical dislocation. Catheters were removed and placed in a sterile microcentrifuge tube containing PBS and vortexed at high speed for 3 min. 25 µl of serial dilution bacteria were plated on TSA and incubated overnight at 37 °C for colony formation units (CFUs) count. Sample size calculation has been carried out using the IVS program. No criteria were established for excluding animals. Confounders were not controlled. Statistical significance differences were determined with one-way ANOVA followed by Dunn’s multiple comparison test: **P < 0.01, ***P < 0.001.

### Ethics statement

All animal studies were reviewed and approved by the Comité de Ética para la Experimentación Animal (CEEA) of the Universidad de Navarra (approved protocol 114-17). Work was carried out at CIMA (ES312010000132) under the principles and guidelines described in European Directive 2010/63/EU for the protection of animals used for experimental purposes.

## Supplementary information

Supplementary Information
