# Supplementary material for: Antibiofilm activity of flavonoids on staphylococcal biofilms through targeting BAP amyloids
Source: Sci Rep. 2020 Nov 3;10:18968. doi: 10.1038/s41598-020-75929-2 (PMC7641273; doi:10.1038/s41598-020-75929-2)
Supplement: Supplementary file 1 — Supplementary Information [file 41598_2020_75929_MOESM1_ESM.pdf]

Supplementary Material

Antibiofilm activity of flavonoids on *staphylococcal* biofilms through targeting BAP amyloids.

Leticia Matilla-Cuenca[1], Carmen Gil[2], Sergio Cuesta[1], Beatriz Rapún-Araiz[2], Miglė Žiemytė[3], Alex Mira[3], Iñigo Lasa[2], Jaione Valle[1*]

[1]Instituto de Agrobiotecnología (IDAB). CSIC-UPNA-Gobierno de Navarra. Avenida Pamplona 123. Mutilva-31192, Spain

[2]Navarrabiomed-Universidad Pública de Navarra-Departamento de Salud, IDISNA, Pamplona-31008, Navarra, Spain

[3]Genomics and Health Department, FISABIO Foundation, 46020 Valencia, Spain

** Corresponding author: jaione.valle@csic.es*

EGCG

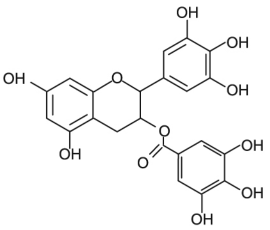

Quercetin (QC)

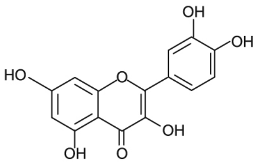

Myricetin (MC)

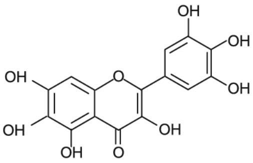

Baicalein (BC)

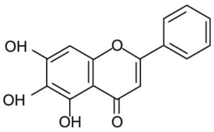

Scutellarein (SC)

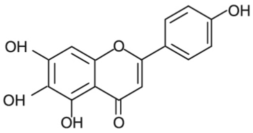

Figure S1: Structures of the polyphenolic compounds used in this study

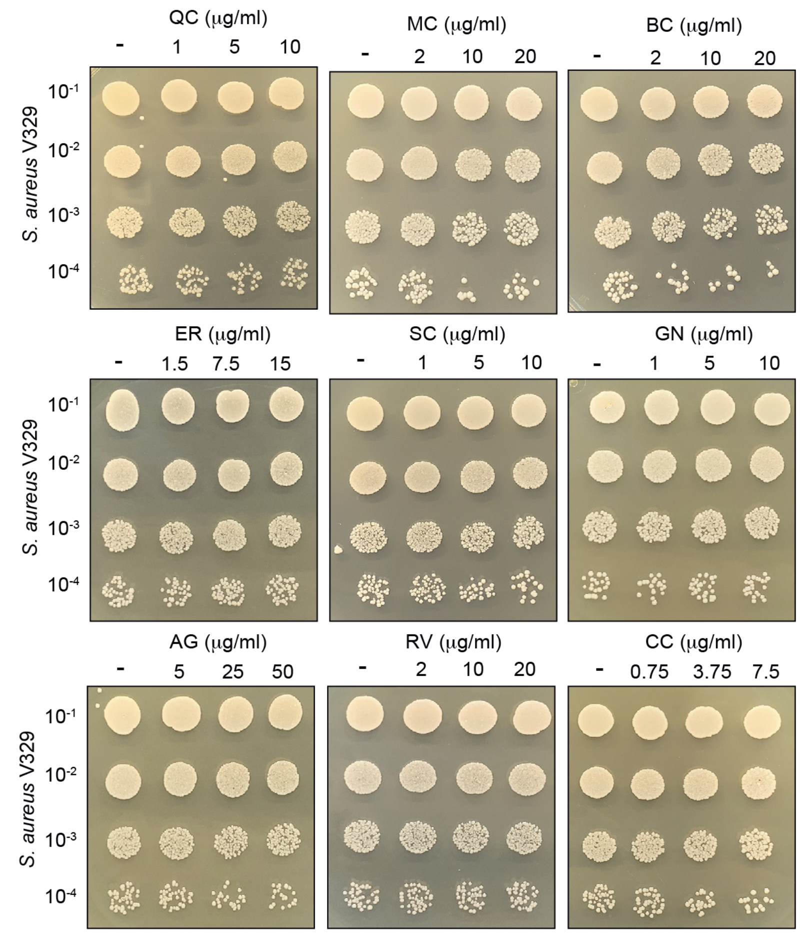

Figure S2. Bacterial growth on TSA medium. *S. aureus* was grown overnight in TSB-glu with sub-MICs dosages of the polyphenols. Serial dilutions were spotted on agar plates and were incubated at 37 ºC.

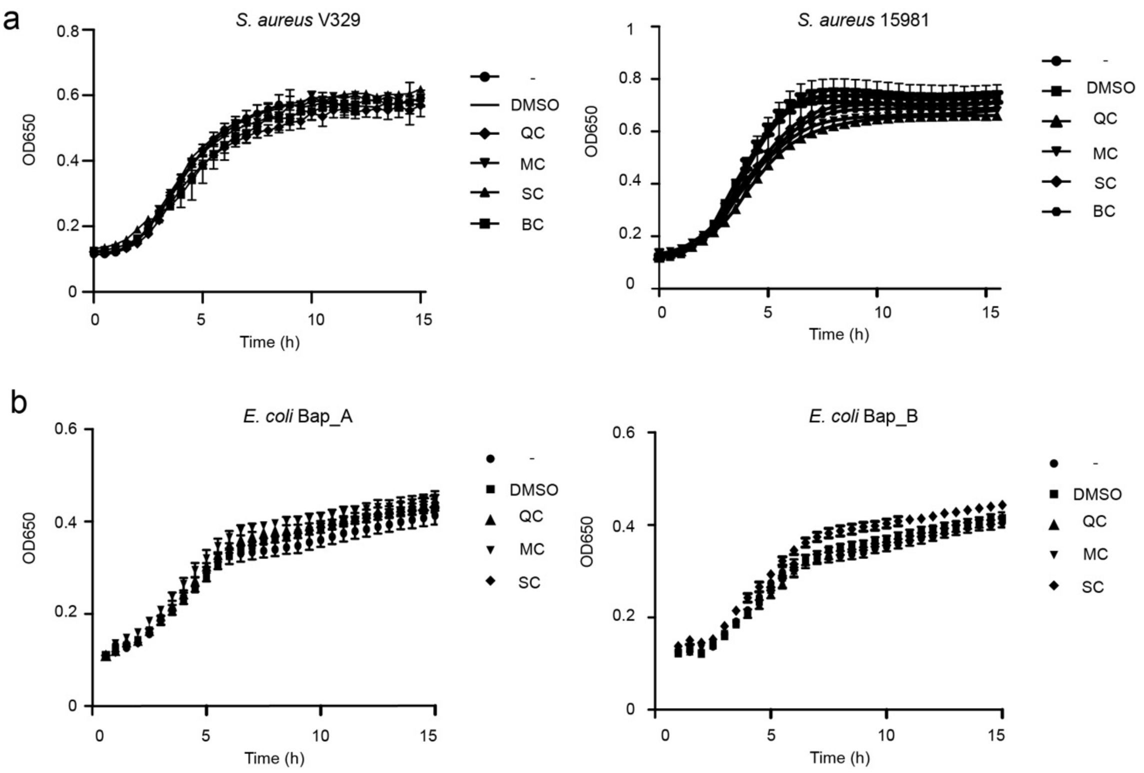

Figure S3: a) Growth curves of *S. aureus* V329 and 15981 strains in TSB-glu medium (-), and medium supplemented with DMSO 2% and MBIC of polyphenols. b) Growth curves of *E. coli* VS39 expressing the Bap_A and Bap_B in LB medium (-), and medium supplemented with DMSO 2% and MBIC of polyphenols. MBIC were used as 10 µg/ml for quercetin (QC), 10 µg/ml for myricetin (MC) and 5 µg/ml for scutellarein (SC), 10 µg/ml for baicalein (BC). Average and SD of three assays are represented.

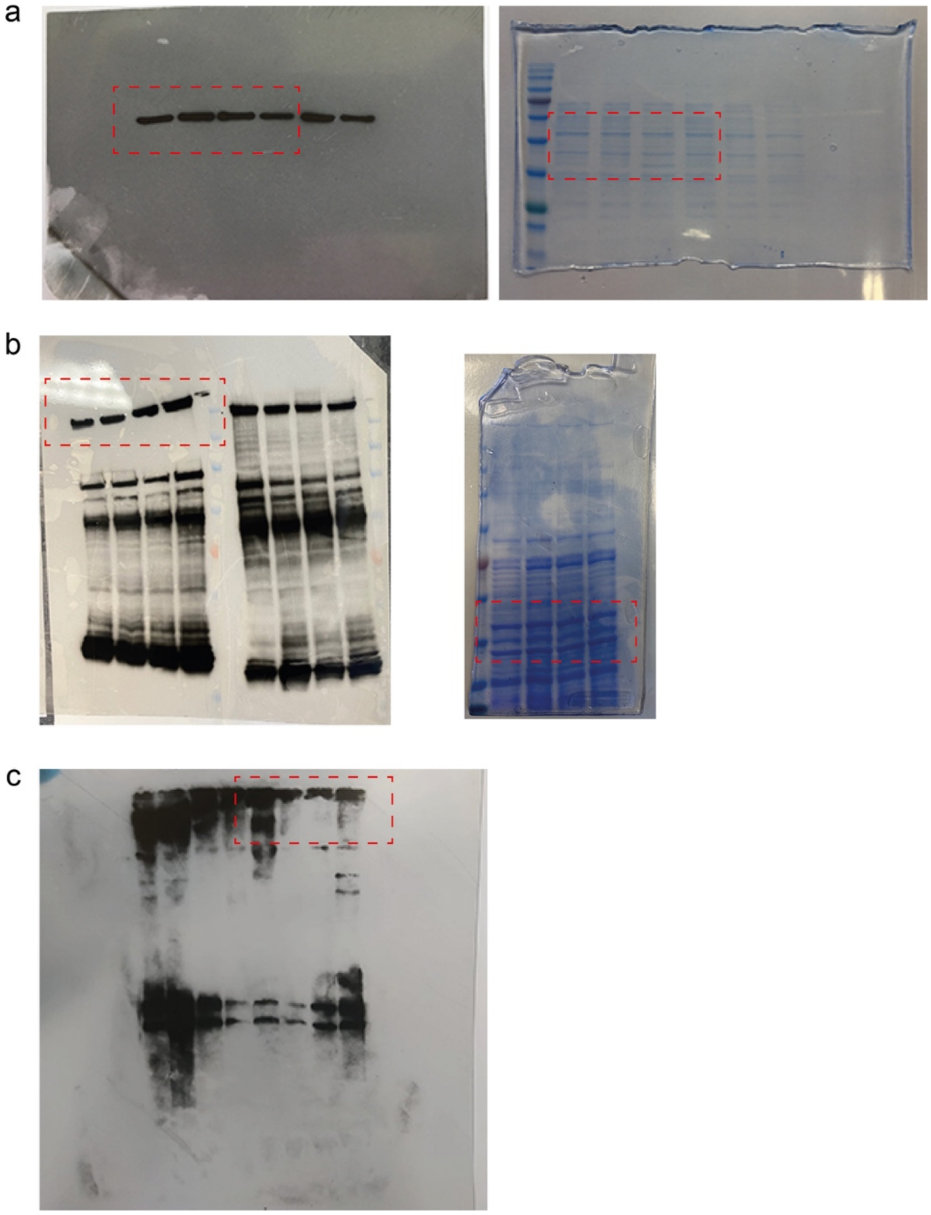

Figure S4. Full-length gels and blots shown in figure 3. a) GFP protein levels of *S. aureus* V329 with pCN52-Pbap:GFP plasmid in presence of MBICs of polyphenols. b) Bap protein levels of *S. aureus* V329 in presence of MBICs of polyphenols. c) Native immunoblotting of cell surface extracts *S. aureus* V329 cultured in presence of MBICs of polyphenols. Cropped gels and blots shown in figure 3 are marked with a red box.
